# Efficacy and safety of percutaneous microwave coagulation therapy followed by ^125^I seed brachytherapy for VX2 hepatic tumors in a rabbit model

**DOI:** 10.3892/etm.2013.1088

**Published:** 2013-04-29

**Authors:** DONG-SHENG YU, HONG CHANG, CHRISTOF-MATTHIAS SOMMER, WEI-LI QU, WEN-JIAN XU, REN-JIE YANG, PENG ZHAO

**Affiliations:** 1 Departments of Interventional Medicine, Qingdao University, Qingdao, Shandong 266003, P.R. China;; 2 Pediatrics, The Affiliated Hospital of Qingdao University Medical College, Qingdao University, Qingdao, Shandong 266003, P.R. China;; 3 Department of Diagnostic and Interventional Radiology, University of Heidelberg, Heidelberg D-69120, Germany;; 4 Department of Interventional Therapy, School of Oncology, Peking University, Beijing 100036, P.R. China

**Keywords:** liver cancer, microwave, ^125^I seed, computed tomography, animal model

## Abstract

The aim of this study was to evaluate the efficacy and safety of percutaneous microwave coagulation therapy (PMCT) followed by ^125^I seed brachytherapy for VX2 liver cancer in rabbits. Eighty New Zealand rabbits were injected with suspensions of VX2 tumor cells to create an animal model. The rabbits were randomly divided into 4 groups (n=20); the control, PMCT, ^125^I seed brachytherapy and combination groups. Group A was treated with PMCT at 40 W for 120 sec, group B was treated with ^125^I seed brachytherapy and group C was treated with PMCT followed by ^125^I seed brachytherapy. Group D were not treated and served as the control group. At 21 days after treatment, the rabbits were sacrificed for pathological assessment. The complete tumor necrosis rate was 19 out of 20 tumors (95%) in group C, 6 (30%) in group A, 0 (0%) in group B and 0 (0%) in the control group. The complete tumor necrosis rate was observed to be significantly different between groups C and A, and between groups C and B (P<0.01). No intraheptic metastasis occurred in group C, compared with an incidence of 7 (35%) in group A, 2 (10%) in group B and 20 (100%) in the control group. Between groups C and A, and between groups C and D, the intraheptic metastasis rate was statistically significant (P<0.01). PMCT followed by ^125^I seed brachytherapy increased the rate of carcinoma necrosis and decreased carcinoma metastasis in the VX2 rabbit model. This combined treatment is a safe, effective and minimally invasive therapeutic option for liver cancer.

## Introduction

Hepatocellular carcinoma is the most common solid organ tumor worldwide. China has a high incidence rate, with ∼300,000 mortalities each year (1). Surgery is the method of choice, but remains unsatisfactory due to the high rate of tumor recurrence. Therefore, treatments of liver cancer with a reliable curative effect and that maximize the protection of liver function are sought.

The most commonly offered therapy is transcatheter arterial chemoembolization (TACE) (2–5). However, in patients with advanced cirrhosis and hepatic decompensation, TACE is contraindicated since the ischemic damage associated with embolization may lead to a rapid decline in liver function with worsening encephalopathy, increased ascites and, potentially, fatality (6–8).

Currently, percutanous microwave coagulation therapy (PMCT) as a liver-directed therapy, offers the potential for extended survival in patients with advanced hepatocellular carcinoma (9–11). However, PMCT success may also be limited by the presence of large portal or hepatic vein branches adjacent to the tumor. The flowing blood may act as a heat sink and limits the ability to heat the tissue to a sufficient temperature. Therefore, PMCT occasionally does not completely kill tumor cells. Another newly developed local treatment, ^125^I seed brachytherapy (12–14), delivers low-dose brachytherapy to the tumor. This treatment is also contraindicated since radiotherapy does not completely kill hypoxic tumor cells.

The purpose of the current study was to evaluate the efficacy and safety of PMCT followed by ^125^I seed brachytherapy for VX2 liver cancer in rabbits, to overcome the respective limitations of the two types of treatment and to explore a novel combined method of treatment for liver cancer.

## Materials and methods

### Establishment of the animal models and tumor growing techniques

Ninety-six New Zealand white rabbits (age, 3–4 months; weight, 3.1–3.6 kg) were used for the experiment. They were provided by Animal Laboratory of the Chinese Air Force General Hospital (Beijing, China). This study was carried out in strict accordance with the recommendations in the Guide for the Care and Use of Laboratory Animals of the National Institutes of Health of China. The animal use protocol was reviewed and approved by the Institutional Animal Care and Use Committee (IACUC) of Qingdao University (Qingdao, China).

In this study, the VX2 carcinoma was maintained via serial transplantation into the hind limb muscle of the New Zealand white rabbit. Following implantation, the tumor enlarged rapidly. In order to reduce the potential difference of tumor growth, tumor tissues were obtained from the same tumor donor rabbit and fresh VX2 tumor tissue was obtained from the same lateral thigh muscle of the New Zealand white rabbit.

For preparation of a VX2 tumor cell suspension, the VX2 tumor was stripped aseptically, mechanically homogenized, filtered through iron mesh with 0.08 mm^2^ pores and centrifuged at 2,000 rpm for 10 min (Centrifuge 5702, Eppendorf, Hong Kong, China). Finally, the viable cells were adjusted to a concentration of 1×10^7^ cells/ml.

Using computed tomography (CT)-guidance, 0.2 ml VX2 tumor cell suspension was percutaneously injected into the center of the lobe of the liver of rabbits slowly using an 18G needle under i.v. anesthesia. With B-ultrasound monitoring the liver of each rabbit, we chose the rabbits with only one tumor implanted in the left liver lobe and its diameter was measured as 2 cm within 14 to 26 days after tumor implantation. Contrast-enhanced CT and MRI were performed to detect the necrotic region of the tumor.

Of the 96 rabbits, 80 (in which the tumor diameter reached 2 cm) were randomly divided into 4 groups (each n=20). The rabbits in group A were treated with CT-guided PMCT at 40 W for 120 sec. The rabbits in group B were treated with CT-guided ^125^I seed brachytherapy (0.5 mCi, range, 60 Gy). The rabbits in group C (combination therapy) were treated with percutaneous microwave coagulation therapy followed by ^125^I seed brachytherapy. The rabbits in the control group (group D) were not treated. At 14 days after surgery, the rabbits were sacrificed for pathological assessment.

### Pretreatment tumor location

Spiral CT scanning conditions were as follows: 120 kV, 200 mA, FOV 14×14 cm, slice thickness 3 mm and spacing 3 mm. The selection of Ultravist^®^ was 300 mg/ml, the dose of contrast was 7 ml, the speed of injection was 3 ml/sec. The animals were anesthetized following the first liver CT scan delay of 10–12 sec, and an early arterial and portal venous phase enhanced scan was conducted to determine the location of the tumor after 40–50 sec. It is extremely important to detect the necrosis of the tumor prior to treatment in order to increase the accuracy of this experiment. In each group prior to treatment, MRI diffusion imaging was also used.

### Microwave coagulation treatment

Group A (the microwave treatment group) consisted of 20 rabbits. Following routine skin preparation, local disinfection and anesthesia, a CT scan was applied to determine the location of the tumor. With a CT-guided microwave coagulation antenna (diameter 1.6 mm) implanted into the tumor center, PMCT was performed with an output power of 40 W for 2 min. The microwave therapeutic instrument and microwave radiation antenna were made by Nanjing Microwave Electronics Research Institute and Nanjing Kia Microwave Technology Ltd. (Nanjing, China).

### ^125^I seed brachytherapy

For group B (^125^I seed brachytherapy group) we used a treatment planning system (TPS) system combined with the classic formula design. The plan for ^125^I seed implantation was designed with the guidance of three experienced doctors from the Department of Radiation Oncology (Beijing Cancer Hospital, Beijing, China). The skin surface of the rabbits was placed on a particle locator (Locator plexiglass; Ningbo Jaco Pharmaceuticals Co. Ltd., Zhejiang, China; hole spacing, 0.5 cm).

CT-guided seed implantation was conducted using a percutaneous locator which pierced the liver around the tumor, to allow the implantation of seeds according to the designed radiation treatment plan. Each rabbit was implanted with 12 ^125^I seeds (three layers, each layer 4 seeds). The radiation dose of each seed was 0.5 mCi. The tumor peripheral prescription dose was 60 Gy. Two days after treatment, plain film of the abdomen (KUB) was administered to the rabbits to determine whether the ^125^I seeds had migrated or not. The ^125^I seed implantation instrument and ^125^I seeds were provided by Ningbo Jaco Pharmaceuticals Co. Ltd. (Ningbo, China).

### Combination treatment (PMCT followed by ^125^I seed brachy-therapy)

Group C were treated with CT-guided percutaneous microwave therapy of the VX2 carcinoma of the rabbits in the same manner as in group A, and ^125^I seed implantation was performed in the same manner as in group B.

### Pathological assessment

At 21 days after treatment, the rabbits were sacrificed by means of Sumianxin II overdose (produced by the Military Veterinary Academy of Medical Sciences Institute, Changchun, China) at 2 ml/kg body weight. The rabbits were fixed in a supine position by abdominal longitudinal midline incision of the upper abdominal skin and local disinfection was performed. Two experienced pathologists (Ji Xiang Rui and Peng Zhao) conducted an assessment with the naked eye on the extent of the tumor and adjacent liver, stomach and intestine, and changes in the gallbladder, diaphragm and skin tissue. Histopathological examination included cross-sectional hematoxylin and eosin (H&E) staining, with a slice thickness of 5 *μ*m, where the pathologist used a light microscope to assess the tumor necrosis rate and the presence of intrahepatic metastasis.

The injuries to the adjacent tissues of the therapeutic region were classified from grade 0 to grade III (grade 0, damage; grade I, mild injury to 1/3 the thickness of the tissue; grade II, moderate injury to 2/3 the thickness of the tissue; grade III, severe injury to the full thickness of the tissue).

### Statistical analysis

Data are presented as the mean ± SD. Gross tumor volumes at different time points were compared by Student’s t-test. Statistical analyses were performed using SPSS 11.0 software (SPSS, Inc., Chicago, IL, USA). P<0.05 was considered to indicate a statistically signifcant result.

## Results

### VX2 hepatic tumor in a rabbit model

Ninety-six New Zealand white rabbits were used to establish the animal model. Eighty of the rabbits developed primary tumors, a success rate of 83.33%.

### CT and MRI findings

**Figure 1.**
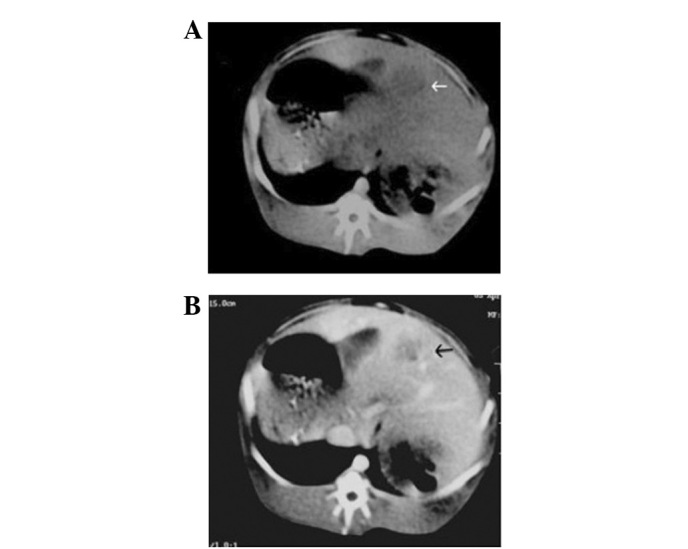
Three weeks after VX2 cell implantation, (A) the computed tomography (CT) plain scanning image demonstrated a tumor (diameter 2 cm) with low density (white arrow) in the liver of an experimental rabbit and (B) CT enhancement scanning image demonstrated the central region of the tumor without intensification but the surrounding region of the tumor intensification

**Figure 2.**
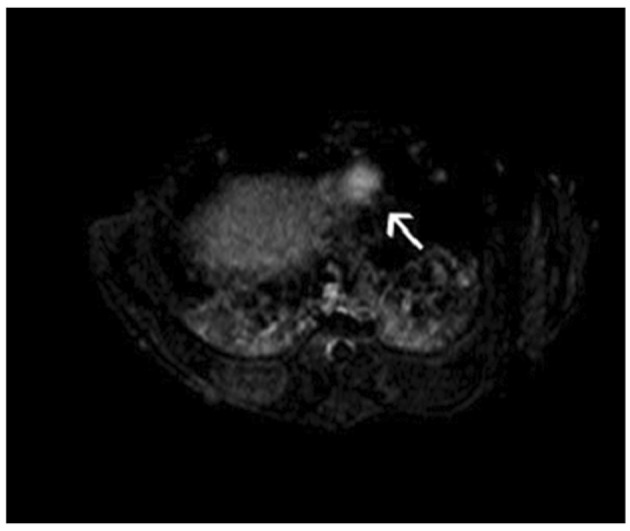
MRI showed a high-intensity signal for the VX2 tumor (white arrow) on diffusion images, allowing clear visualization of the boundary. No necrosis with low-intensity signals was detected for the VX2 tumor prior to treatment.

With B-ultrasound monitoring the liver of each rabbit, we chose the rabbits with only one tumor implanted in the left liver lobe and its diameter was measured as 2 cm within 14 to 26 days after tumor implantation. CT plain scanning images revealed a tumor (diameter 2 cm) with low density in the liver of an experimental rabbit (Fig. 1A) and CT enhancement scanning images (Fig. 1B) demonstrated that the tumor had a low-density central region without intensification but the surrounding region of the tumor intensification. MRI showed the VX2 tumor with a high-intensity signal by diffusion imaging, allowing clear visualization of the boundary. No clear signs of central necrosis were shown on B-ultrasound when the tumor sizes were almost 20 mm. This was also verified by CT contrast imaging (Fig. 1B) and MRI diffusion imaging (Fig. 2).

**Figure 3.**
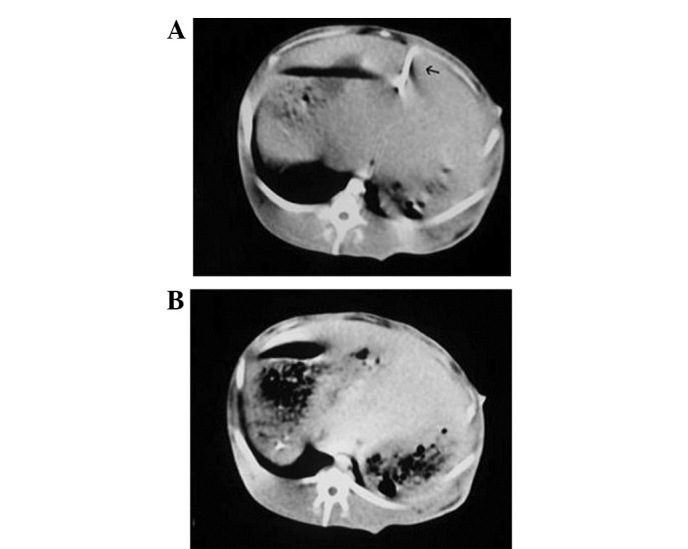
(A) Computed tomography (CT) plain scanning image demonstrating the microwave ablation needle in the center of the tumor (black arrow) during the computed tomography CT-guided percutaneous microwave coagulation therapy (PMCT) at 40 W for 120 sec. (B) Small bubbles were observed in the region of the tumor five minutes after the ablation on the CT plain scanning image.

CT plain scanning images revealed the microwave ablation needle in the center of the tumor during the CT-guided PMCT at 40 W for 120 sec (Fig. 3A). Small bubbles were observed in the region of the tumor five minutes after the ablation on CT plain scanning images (Fig. 3B).

**Figure 4.**
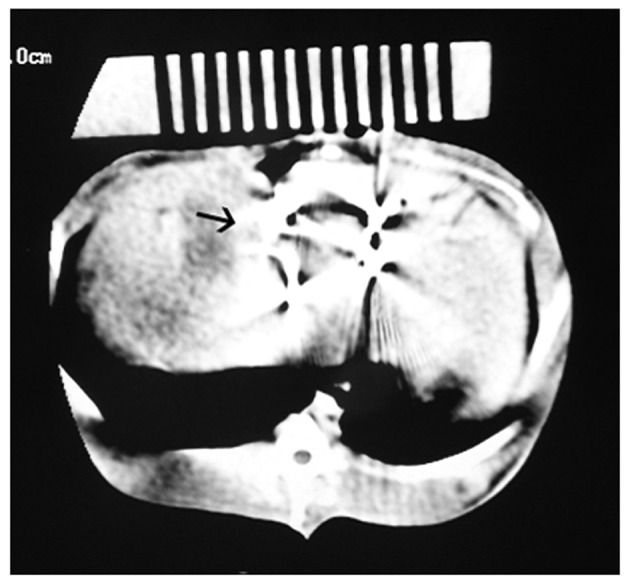
Computed tomography (CT) plain scanning images demonstrate that the ^125^I seed seeds (black arrow) were implanted around the tumor during the CT-guided ^125^I seed brachytherapy (0.5 mCi, range dose, 60 Gy).

CT plain scanning images demonstrated that the ^125^I seeds were implanted around the tumor during the CT-guided ^125^I seed brachytherapy (0.5 mCi, range dose, 60 Gy; Fig. 4).

### Histopathological findings

**Figure 5.**
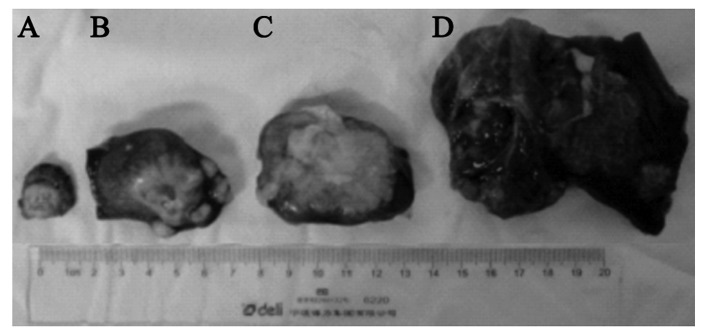
Gross specimens show that the tumor in (A) the group treated with combined PMCT followed by ^125^I seed brachytherapy (group C) was the smallest among the four groups with completely necrosis and no liver metastasis compared with (B) PMCT (group A) and (C) ^125^I seed brachytherapy (group B). The tumor in (D) control group (group D) was the largest in size, with liver metastasis. PMCT, percutaneous microwave coagulation therapy.

Gross specimens show that the tumors treated with combined PMCT following ^125^I seed brachytherapy (group C) were the smallest tumors of the four groups, although tumor size is not an assessment criterion in this experiment, with completely necrosis and no liver metastasis compared with ^125^I seed brachytherapy (group B) and PMCT (group A). The tumors in the control group (group D) were the largest in size and liver metastasis was also present in this group (Fig. 5).

PMCT followed by ^125^I seed brachytherapy (group C) resulted in complete necrosis in 19 of 20 (95%) tumors, compared with 6 of 20 (30%) tumors treated with PMCT alone (group A) and 0 of 20 (0%) tumors treated with ^125^I seed brachytherapy alone (group B; P<0.01).

No liver metastasis occurred in the rabbits which received the combined treatment (group C), whereas metastasis was observed in 7 of 20 (35%) rabbits treated with PMCT alone (group A), 2 of 20 (10%) rabbits treated with ^125^I seed brachytherapy alone (group B) and all 20 rabbits (100%) in the control group (group D).

In the microwave treatment group (group A), there were two cases (2/20) in which the treatment area was adjacent to the gallbladder and grade I mild injury was present. No severe necrotic changes were observed in the adjacent tissues of the therapeutic region on the cutis, stomach, bowel, gallbladder or diaphragm of the rabbits in groups A, B and C.

The results showed that the tumor necrosis rate of group C was significantly higher than those of groups A and B, which indicates that combination therapy increases the tumor necrosis rate. PMCT followed by ^125^I seed brachytherapy is a safe, effective and minimally invasive therapeutic option for liver cancer.

## Discussion

PMCT followed by ^125^I seed brachytherapy is a safe, minimally invasive and promising therapeutic option for liver cancer due to the eradication of local tumor cells.

PMCT appears to be a useful and safe treatment, particularly for cases of superficial hepatocellular carcinoma. Microwave irradiation creates an ablation area around the needle in a columnar or round shape, depending on the type of needle used and the generating power. However, similar to other thermal methods, such as radio frequency (RF) ablation for local tumor treatment (15,16), tumor size and the presence of large (≥3 mm) abutting vessels significantly affects the outcome of the procedure. Another limitation of microwave ablation is the lesion location. The treatment of lesions located along the liver surface, particularly in proximity to the gastrointestinal tract, or adjacent to the porta hepatis or the gallbladder, are at risk of major complications (17).

CT-guided permanent brachytherapy was initially used for treating liver malignancies (18,19), recurrent rectal carcinoma and spinal metastatic and primary paraspinal malignancies (20). This novel technique ensures protracted cell killing over a period of several months via targeted delivery of high-dose radiation. The advantages of this technique are as follows; i) it is minimally invasive, ii) dose distribution may be accurately predicted, iii) continuous irradiation increases the likelihood of damaging malignant cells in a vulnerable phase of the cell cycle and iv) the incidence rate of acute adverse effects is low. However, radiotherapy does not completely kill hypoxic tumor cells, but does have cytoreductive effects.

In the present study, complete tumor necrosis was achieved in 19 of 20 (95%) tumors and no intraheptic metastasis was present in the rabbits in the combined PMCT and ^125^I seed brachytherapy group. The results show that combined PMCT followed by ^125^I seed brachytherapy to the liver cancer has certain advantages. Although PMCT is a viable method for the treatment of cancer, in the majority of cases, the tumor blood flow removes part of the heat from the microwaves. Since the shape of the microwave ablation region is not round, residual tumor tissue is often observed at the tumor margin. To eliminate these unfavorable treatment factors, combined PMCT should be followed by ^125^I seed brachytherapy, which may provide improved local control of the tumor. To the best of our knowledge, combined PMCT followed by ^125^I seed brachytherapy to the VX2 rabbit liver cancer has never previously been documented.

The VX2 tumor is a squamous carcinoma from a virus-induced papilloma. However, the behavior of VX2 tumors is similar to that of the primary tumor. The biological characteristics of VX2 tumors and liver cancer cells are similar, so VX2 tumors are widely used to study human hepatocellular carcinoma.

Although PMCT and ^125^I seed brachytherapy have a low likelihood of causing complications (19–22), care should be taken to avoid possible complications following ^125^I seed brachytherapy and microwave combination therapy. We studied irreversible injury changes of the tissues adjacent to the therapeutic region, not reversible injury. Therefore, tissue congestion, edema and reversible tissue damage, such as the two cases of gallbladder grade I mild injury, may be ignored in this experiment.

The main purpose of this experiment was to verify if the actions of PMCT followed by ^125^I seed brachytherapy for VX2 hepatic tumors in a rabbit model are synergistic. Therefore, in this experiment, the elevated intrahepatic metastasis rate of the microwave group and the reduced complete tumor necrosis rate of the ^125^I seed brachytherapy group do not have universal significance. The complete tumor necrosis rate of PMCT followed by ^125^I seed brachytherapy is up to 95%, however, the limitations of the animal models require consideration. For clinical applications, further study required.

For example, how to combine the thermal field of the PMCT with the radiation field of ^125^I Seeds effectively in some liver cancer patients with Child-Pugh liver function of score C (23). It is beneficial to combine ablation with radiation for the treatment of tumors (24). PMCT followed by ^125^I seed brachytherapy is a safe, effective and promising minimally invasive therapeutic option for liver cancer.

## Figures and Tables

**Figure 6.**
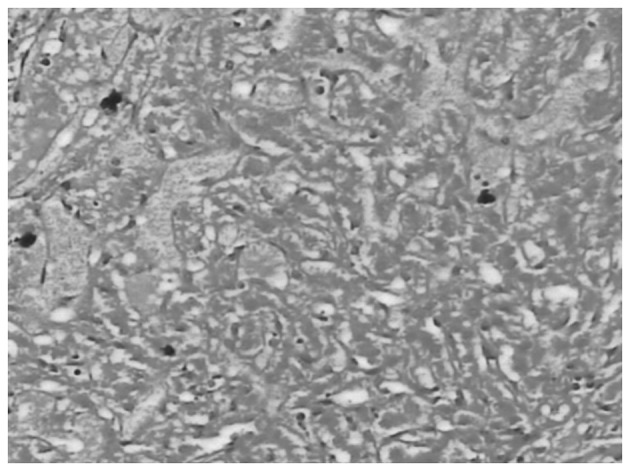
Representative H&E histology of complete necrosis of the region of tumor cells treated with percutaneous microwave coagulation therapy followed by ^125^I seed brachytherapy (group C; magnification, ×200). H&E, hematoxylin and eosin.
